# CMV on surfaces in homes with young children: results of PCR and viral culture testing

**DOI:** 10.1186/s12879-018-3318-z

**Published:** 2018-08-13

**Authors:** Minal M. Amin, Jennifer D. Stowell, William Hendley, Philip Garcia, D. Scott Schmid, Michael J. Cannon, Sheila C. Dollard

**Affiliations:** 1 Centers for Disease Control and Prevention (CDC), 1600 Clifton Road NE, Atlanta, GA 30329 USA; 2 Emory University, Atlanta, USA

**Keywords:** Cytomegalovirus, Viral shedding, Infectious virus, Transmission

## Abstract

**Background:**

Caring for young children is a known risk factor for cytomegalovirus (CMV) infection mainly through exposure to their saliva and urine. In a previous study, 36 CMV-seropositive children 2 mo. to 4 years old were categorized as CMV shedders (*n* = 23) or non-shedders (*n* = 13) based on detection of CMV DNA in their saliva and urine. The current study evaluated the presence of CMV on surfaces in homes of the children.

**Methods:**

Study staff made 4 visits to homes of the 36 enrolled children over 100 days. Saliva was collected by swabbing the mouth and urine was collected on filter paper inserted into diapers. In addition, five surface specimens were collected: three in contact with children’s saliva (spoon, child’s cheek, washcloth) and two in contact with children’s urine (diaper changing table, mother’s hand). Samples were tested by PCR and viral culture to quantify the presence of CMV DNA and viable virus.

**Results:**

A total of 654 surface samples from 36 homes were tested; 136 were CMV DNA positive, 122 of which (90%) were in homes of the children shedding CMV (*p* < 0.001). Saliva–associated samples were more often CMV positive with higher viral loads than urine-associated samples. The higher the CMV viral load of the child in the home, the more home surfaces that were PCR positive (*p* = 0.01) and viral culture positive (*p* = 0.05).

**Conclusions:**

The main source for CMV on surfaces in homes was saliva from the child in the home. Higher CMV viral loads shed by children correlated with more viable virus on surfaces which could potentially contribute to viral transmission.

## Background

Cytomegalovirus (CMV) infection is the most common cause of congenital viral infection in developed countries. Primary infection and reinfection of adults occurs at higher rates among those in contact with young children in the home, daycare centers and schools [1–3]. Children appear to be an important source for CMV infection in all of these environments [4, 5]. Many studies have tracked CMV shedding in young children showing that virus is shed in the saliva and urine of half or more of young seropositive children, and that rates of shedding peak at 1–2 years of age [5–8]. For women of reproductive age, contact with young children poses the greatest risk for transmission [9]. Parents with children shedding CMV have 10 times the seroconversion rate of parents whose children are not shedding CMV [10]. Although direct contact with child saliva and urine is a well-known transmission risk, very few studies have tested surfaces that contact children’s fluids for their potential to harbor CMV and contribute to transmission.

In this study, we tested for the presence of CMV by quantitative PCR and viral culture on samples taken from surfaces in homes where young CMV-seropositive children lived to better understand potential routes of CMV transmission beyond direct contact with the saliva and urine of shedding children.

## Methods

### Study population and specimen collection

In a previous study we enrolled 36 healthy, CMV-seropositive children 2 mo. to 4 years old (mean age 17 months) that were still in diapers from regional daycare centers and pediatrician’s offices in the metropolitan Atlanta area. None of the children had chronic medical conditions or had been diagnosed with congenital CMV infection [11]. Saliva was collected by using a sterile oral swab as previously described [11]. Urine collection was done by providing disposable diapers to parents that had strips of filter paper inserted in the front panel (Whatman 903 paper used in the U.S. newborn screening (NBS) program). Previous studies have reported using filter paper to collect urine and test for CMV [12, 13]. Urine-soaked inserts were removed with tweezers by the parents, air-dried 4 to 12 h on a clean nonporous surface, placed in Ziploc bags and stored in the refrigerator until the next staff visit, usually within 24 h. Ultraviolet light causes urine on the filter paper to fluoresce to verify its presence. One 6 mm punch was taken and processed for DNA extraction using the same method used to test blood on filter paper for CMV [14].

Surface specimens were collected at 4 times by study staff at the same visits where saliva and urine were collected from children. At each visit, 5 surface specimens were collected using sterile cotton-tipped swabs (Fisher Scientific, Pittsburg, PA) pre-moistened with phosphate buffered saline (PBS): (1) Spoon; spoon shortly after removal from child’s mouth, (2) Cheek; child’s cheek area where there was visible moisture, (3) Washcloth; washcloth shortly after the mother used it to wipe the child’s mouth, (4) Hands; the palm of the mother’s hand immediately after a diaper change, (5) Changing area; changing table immediately after a diaper change. Swabs were immediately immersed in 1 ml viral transport media (VTM, Fisher Scientific, Pittsburg, PA) in 15 ml tubes, placed on ice and transported to the CDC laboratory for processing, the same day in most cases.

### CMV detection by polymerase chain reaction (PCR) and viral culture

Swabs in VTM were removed to a syringe and centrifuged 10 min at 2000 rpm to extract liquid which was mixed back with the VTM. For PCR testing, 100 μl was removed and mixed with 100 μl of Quick Extract buffer (EpiCenter, Madison, WI), incubated 1 h at 56 °C, then 100 °C for 3 min, then cooled on ice. The extract was used for Taqman-based PCR targeting the CMV gB gene [15] with commercially standardized human CMV DNA quantitated with digital PCR by Advanced Biotechnologies, Inc. (Eldersburg, MD) included on every PCR plate. NB: The international CMV PCR standard provided by the World Health Organization from a manufacturer in England has been logistically difficult for U.S. residents to purchase. For analysis low viral load was defined as < 10^4^ copies/ml, medium viral load as 10^4^–10^6^ copies/ml, and high viral load as > 10^6^ copies/ml.

For viral culture, the remaining VTM (about 0.9 ml) was added to a T-25 tissue culture flask containing human lung fibroblast cells (ATCC, Manassas, VA, USA) from which media had been removed. Following 1 h adsorption, 5 ml of fresh culture medium with antibiotics was added to the flask. Cultures were observed for cytopathic effect twice per week for 3 weeks. Urine samples were dried on filter paper and thus not usable for culture.

### Statistical analysis

Associations were examined using SAS version 9.3 (Cary, NC). We calculated *P*-values for proportions by using the chi-square or Fisher’s exact tests as appropriate. When comparing viral loads or antibody titers, we used the Cochran-Mantel-Haenszel test and the Wilcoxon rank-sum test.

## Results

### Specimens collected and presence of CMV by PCR

**Fig. 1:**
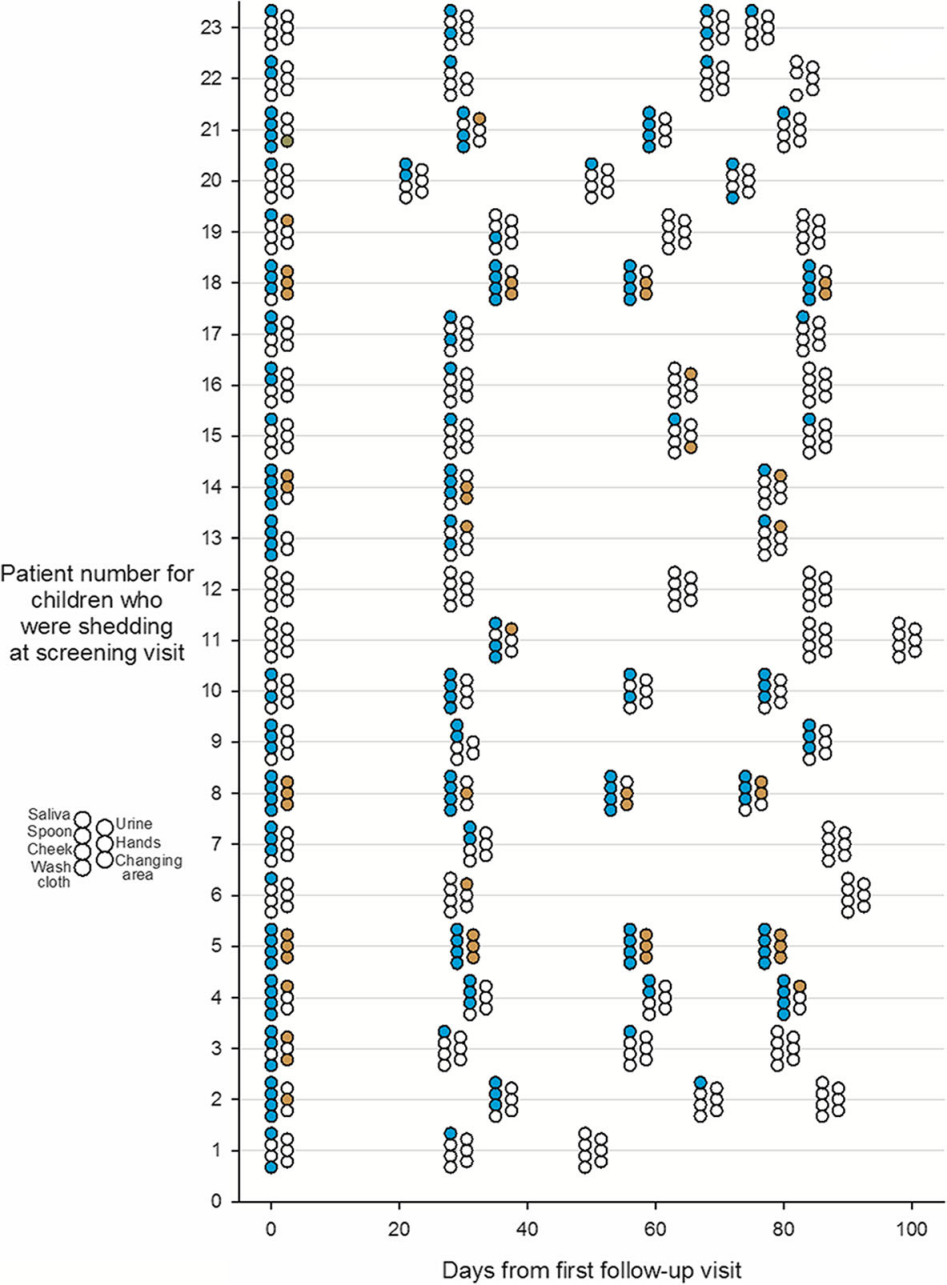
CMV PCR results on specimens collected in homes of children shedding CMV. Various specimens were collected at four follow-up visits to homes over 100 days. Children living in the homes were shedding CMV when tested at the screening visit which was prior to the first follow-up and is not shown on the graph. Colored circles signify a positive result, open circles a negative result. Blue color is for saliva and its contact surfaces, yellow is for urine and its contact surfaces. At each visit, up to seven specimens were collected, as shown to the left of the chart

**Fig. 2:**
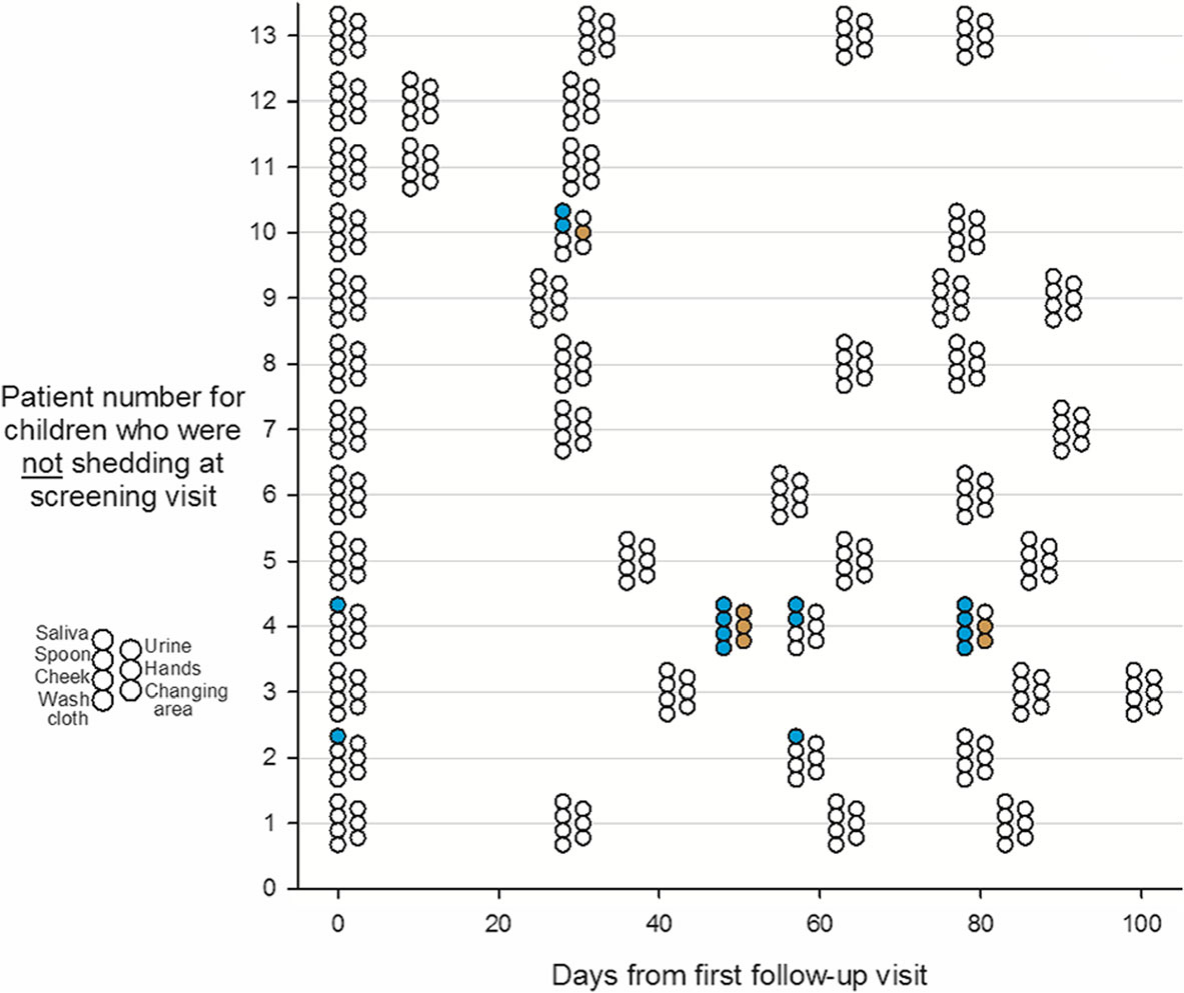
CMV PCR results on specimens collected in homes of children not shedding CMV. Various specimens were collected at four follow-up visits to homes over 100 days. Children living in the homes were not shedding CMV when tested at the screening visit which was prior to the first follow-up and is not shown on the graph. Colored circles signify a positive result, open circles a negative result. Blue color is for saliva and its contact surfaces, yellow is for urine and its contact surfaces. At each visit, up to seven specimens were collected, as shown to the left of the chart

A total of 913 specimens were collected over 4 visits from 36 children in 35 households; 259 specimens were from children and 654 from surfaces. Figure 1 shows PCR results for all samples collected at all visits for the children who were shedding CMV at enrollment, and Fig. 2 shows the same for children who were not shedding CMV at enrollment. The enrollment visit was prior to day 0 and is not shown in Figs. 1 and 2. CMV DNA was detected in 59% (502/846) of saliva specimens and 20% (167/844) of urine specimens [16]. Among surface specimens, 136/654 (21%) were CMV DNA positive, 90% of which (123/136) were collected from homes of the shedding children (*p* < 0.001) and 10% from the homes of non-shedding children which was mainly from one home where the child began shedding CMV after enrollment.

### CMV viral load

**Fig. 3:**
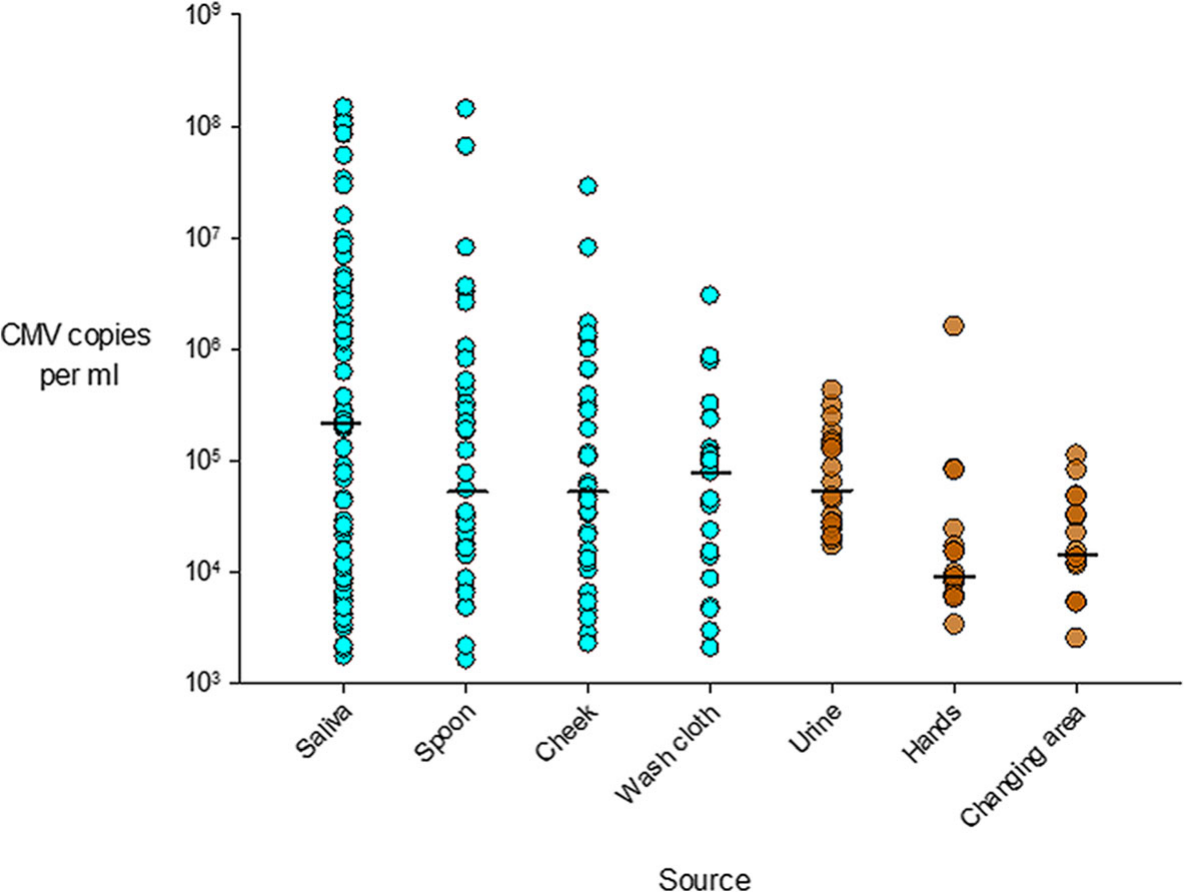
Viral load range for each sample type, horizontal lines represent median viral load values. Blue color is for saliva and its contact surfaces, yellow is for urine and its contact surfaces

**Fig. 4:**
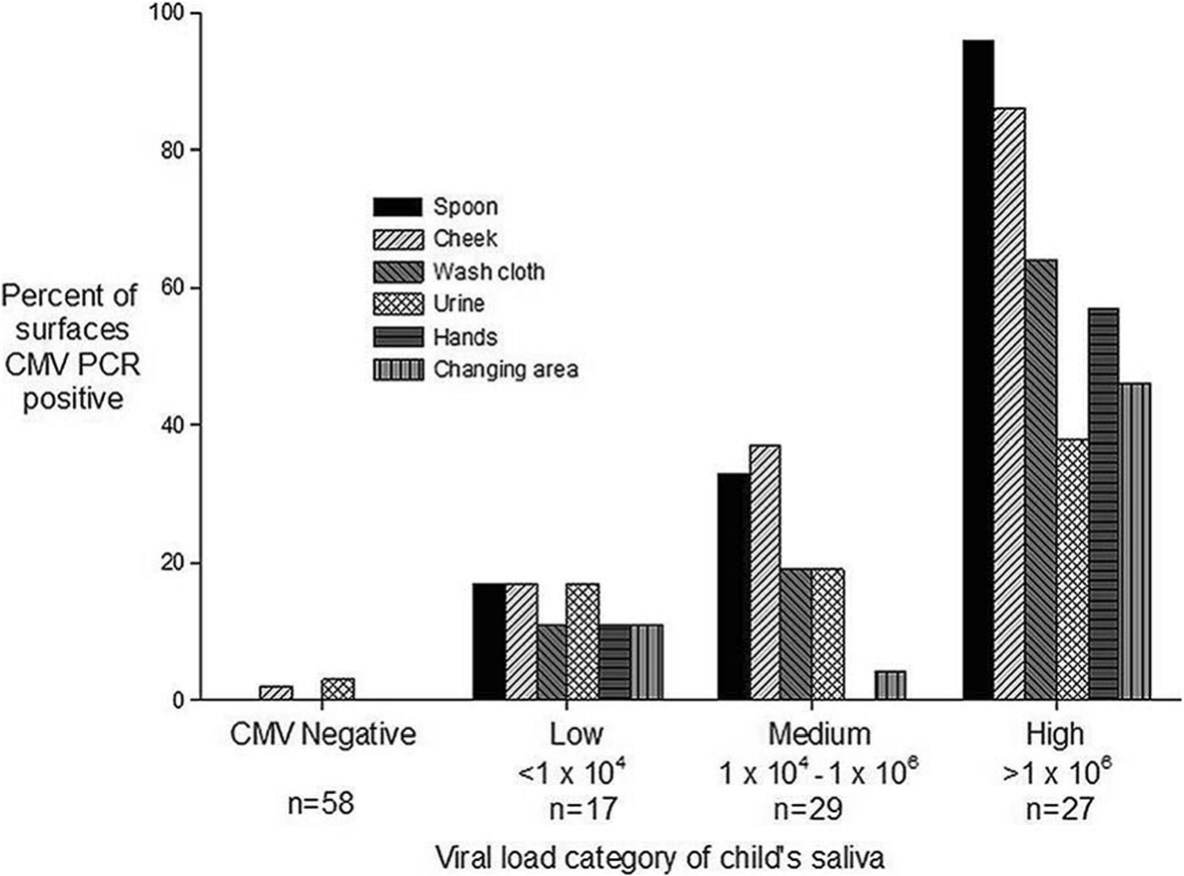
CMV PCR-positivity (%) of the six specimen types that are categorized according to the CMV viral load (low, medium, high) of the saliva from the child living in the home from which the specimens were collected. N = the total number of saliva specimens collected per category

Specimens were CMV PCR positive at a rate of 12–56% depending on the type of specimen (Table 1). Generally, saliva and saliva-associated surfaces samples resulted positive at higher frequency and with higher viral loads than urine and urine-associated surface samples., with statistically significant differences listed in Table 1 and as followssaliva compared to every other source; Spoon and cheek compared to urine, hands and changing area. Mean viral loads were significantly different for the following pairs: Saliva compared to cheek, washcloth, urine, hands, and changing area. Spoon, cheek, washcloth and urine compared to hands and changing area. Median viral loads were significantly different for the following pairs: Saliva, spoon, cheek, washcloth and urine compared to hands and changing area. Figure 3 displays the full range and median viral load (horizontal line) for each specimen type. Median viral loads were similar for saliva and urine; however, saliva-associated surfaces (spoon, cheek, wash cloth) had more of the high viral load specimens compared to urine-associated surfaces (hands, changing area). Figure 4 shows the correlation between increasing CMV viral loads in children’s saliva and increasing portion of surfaces in the home that were CMV-positive (*p* = 0.01) as evidence that the shedding child in the home was the source of CMV in the home. The same three viral load categories shown in Table 2 were used for analysis in Fig. 4 (< 10^4^, 10^4^–10^6^, > 10^6^).

**Table 1:** Results of CMV detection by PCR and cell culture from different sample types (saliva- and urine-associated)

Source	No. positive/total tested^a^	95% confidence interval for proportion	Mean viral load (copies/mL)	Standard deviation (copies/mL)	Median viral load (copies/mL)^b^	Interquartile range, Q1-Q3 (copies/mL)	No. positive/total cultures
Saliva	73/131 (56%)	47–64%	1.15 × 10^7^	3.01 × 10^7^	2.08 × 10^5^	1.07 × 10^4^–3.42 × 10^6^	44/54 (81%)
Spoon	39/131 (30%)	22–38%	5.86 × 10^6^	2.45 × 10^7^	5.47 × 10^4^	1.55 × 10^4^–4.26 × 10^5^	10/20 (50%)
Cheek	38/130 (29%)	22–39%	1.22 × 10^6^	4.76 × 10^6^	5.25 × 10^4^	1.20 × 10^4^–3.80 × 10^5^	4/16 (25%)
Wash cloth	25/131 (19%)	13–27%	2.53 × 10^5^	6.10 × 10^5^	7.83 × 10^4^	1.39 × 10^4^–1.27 × 10^5^	3/12 (25%)
Urine	20/128 (16%)	10–23%	1.05 × 10^5^	1.10 × 10^5^	5.46 × 10^4^	2.57 × 10^4^–1.43 × 10^5^	Not done
Hands	18/131 (14%)	8–21%	4.05 × 10^4^	3.65 × 10^5^	9.12 × 10^3^	6.11 × 10^3^–1.67 × 10^4^	1/14 (7%)
Changing area	16/131 (12%)	7–19%	2.83 × 10^4^	3.01 × 10^4^	1.41 × 10^4^	8.36 × 10^3^–3.96 × 10^4^	1/8 (13%)

^a^Differences in the percentage of PCR-positive results were significant (*P* < 0.05, Cochran-Mantel-Haenszel test) for the following pairs: Saliva compared to every other source; Spoon and cheek compared to urine, hands and changing area. ^b^Median viral loads were significantly different (*P* < 0.05, Wilcoxon rank-sum test) for the following pairs: Saliva, spoon, cheek, washcloth and urine compared to hands and changing area

**Table 2:** Association between PCR viral load and viral culture positivity

CMV Viral Load Category	Culture Positive/Total Cultures
< 1 × 10^4^ copies/mL	7/38 (18%)
1 × 10^4^–1 × 10^6^ copies/mL	31/57 (54%)
> 1 × 10^6^ copies/mL	24/33 (73%)

Test for linear trend for percent positive *p* < 0.001

### Presence of CMV by viral culture

Valid viral culture results were available for 64% (398/626) of specimens processed for culture; 36% failed for technical reasons including poor quality cell sheets (20%), or heavy bacterial contamination (16%) despite the use of antibiotics in culture media. Culture results by specimen type are displayed in Table 1. Most (62/64 = 97%) of the culture-positive specimens were also CMV PCR positive and thus Table 1 lists culture results only for PCR-positive specimens. Spoons from the mouths of children were more often viral culture positive (50%) than other surfaces tested (7–25%) (*p* = 0.05). The higher viral load specimens were saliva and saliva-associated surfaces and were more often positive by viral culture than lower viral load specimens associated with urine. Urine was not amenable to viral culture testing because it was dried on filter paper. High PCR viral load was strongly associated with positive viral culture (Table 2; test for linear trend for percent positive, < 0.001).

## Discussion

Our study evaluated CMV on various home surfaces exposed to saliva and urine of young CMV-seropositive children to better understand potential routes of transmission. The same children were enrolled in a related study that took weekly measurements of their saliva and urine over 100 days to measure CMV shedding [11, 16]. Results of the current study indicate that children in the home were the main source of CMV on home surfaces, and that the child’s saliva was more a source of virus than the child’s urine. This would be expected since urine is generally contained by diapers whereas saliva is not contained by a barrier. Multiple lines of evidence support our findings: 90% of the CMV DNA-positive specimens were found in homes where the shedding children lived and 10% in the homes where non-shedding children lived (mainly in one home where the child began shedding after enrollment), and rates of CMV positivity of home surfaces increased with higher CMV viral loads of the child in the home. Other household members were unlikely sources of surface CMV; none of the enrolled children had siblings under 3 years of age, and adults do not often shed CMV beyond primary infection [5]. Moreover CMV seroprevalence was nearly the same for the mothers of shedders (91%) and non-shedders (92%).

Unique aspects of our study include the focus on surfaces in homes where there are few potential sources of CMV, measurement of wild-type virus, and known CMV sero-status of children. Several studies since the 1980’s have documented high rates of CMV shedding and transmission showing that young children are a main source of CMV transmission, and peak rates of shedding occur at 1–2 years of age [5, 17–21]. The limited number of studies that examined environmental CMV tested surfaces inoculated with laboratory strains of CMV [22, 23], or were conducted in hospital nurseries with multiple potential sources of CMV that detected viable virus on medical equipment [24] or found negligible amounts of CMV on hospital surfaces [25]. One study conducted in a daycare center reported that 4% of surfaces were CMV positive, however, there was no description of surfaces tested or determination of the children’s CMV serostatus [7].

Another useful result of our study is demonstration of the effective use of 903 filter paper to collect and test urine for CMV. The U.S. newborn screening program collects blood on 903 filter paper from a heel stick from virtually all infants. Blood spots are then used to screen for a wide range of birth defects. Our laboratory has previously used newborn blood spots to test for CMV [14], and in the current study we successfully used the same diagnostic methods for urine on filter paper as used for blood spots Other studies have successfully collected child urine on filter paper for CMV testing [12, 13] though not using the same diagnostic methods as used for blood spots. If universal screening for CMV were to be considered, urine collected and dried on 903 filter paper could potentially be processed similarly to DBS by the highly efficient and cost-effective U.S. newborn screening infrastructure. Limitations of our study included the relatively small sample size of 36 children and not testing the fluids of adult household members for possible CMV shedding.

Several studies to date indicate prevention education can reduce CMV infection [26–28]. Our study adds to the growing body of evidence that saliva may be the main vehicle for CMV transmission more than urine and provides evidence that objects with the child’s saliva such as eating utensils can have high viral loads and viable virus that may contribute to transmission.

## Conclusions

The main source for CMV on surfaces in homes was young children living in the home. Surfaces associated with saliva had higher CMV viral loads and more viable virus than surfaces associated urine, therefore saliva is likely more important vehicle for transmission than urine. Better understanding of the specific routes of transmission can lead to prevention messages that are more evidence-based and effective.

## Abbreviations

CMV: Cytomegalovirus
DBS: Dried blood spots
gB: Glycoprotein B
NBS: Newborn screening
PBS: Phosphate buffered saline
PCR: Polymerase chain reaction
VTM: Viral transport medium
